# Treating Nephroblastoma in Rwanda: Using International Society of Pediatric Oncology Guidelines in a Novel Oncologic Care Model

**DOI:** 10.1200/JGO.2015.000067

**Published:** 2016-01-27

**Authors:** Cyprien Shyirambere, Mary Jue Xu, Shekinah Nefreteri Elmore, Temidayo Fadelu, Leana May, Neo Tapela, Denis Gilbert Umuhizi, Frank Regis Uwizeye, Caitlin Driscoll, Clemence Muhayimana, Vedaste Hategekimana, Fidel Rubagumya, Ignace Nzayisenga, Lawrence N. Shulman, Tharcisse Mpunga, Leslie E. Lehmann

**Affiliations:** **Cyprien Shyirambere**, **Neo Tapela**, **Frank Regis Uwizeye**, and **Ignace Nzayisenga**, Inshuti Mu Buzima/Partners in Health Rwanda; **Denis Gilbert Umuhizi**, **Clemence Muhayimana**, **Vedaste Hategekimana**, **Fidel Rubagumya**, and **Tharcisse Mpunga**, Ministry of Health Rwanda, Kigali, Rwanda; **Mary Jue Xu**, **Shekinah Nefreteri Elmore**, **Neo Tapela**, **Lawrence N. Shulman**, and **Leslie E. Lehmann**, Harvard Medical School; **Temidayo Fadelu**, **Neo Tapela**, and **Leslie E. Lehmann**, Brigham and Women’s Hospital; **Leana May** and **Leslie E. Lehmann**, Boston Children’s Hospital; **Lawrence N. Shulman** and **Leslie E. Lehmann**, Dana-Farber Cancer Institute, Boston, MA; and **Caitlin Driscoll**, Icahn School of Medicine at Mount Sinai, New York, NY.

## Abstract

**Purpose:**

Success in treating nephroblastoma in high-income countries has been transferred to some resource-constrained settings; multicenter studies report disease-free survival of greater than 70%. However, few reports present care models with rural-based components, care tasks shifted to internists and pediatricians, and data collection structured for monitoring and evaluation. Here, we report clinical outcomes and protocol compliance for patients with nephroblastoma evaluated at Butaro Cancer Center of Excellence in Rwanda.

**Patients and Methods:**

This retrospective study reports the care of 53 patients evaluated between July 1, 2012, and June 30, 2014. Patients receiving less than half of their chemotherapy at Butaro Cancer Center of Excellence were excluded.

**Results:**

Of the 53 patients included, 9.4% had stage I, 13.2% had stage II, 24.5% had stage III, 26.4% had stage IV, and 5.7% had stage V disease; the remaining 20.8% had unknown stage disease from inadequate work-up or unavailable surgical report. The incidence of neutropenia increased with treatment progression, and the greatest proportion of delays occurred during the surgical referral phase. At the end of the study period, 32.1% of patients (n = 17) remained alive after treatment; 24.5% (n = 13) remained alive while continuing treatment, including one patient with recurrent disease; 30.2% (n = 16) died; and 13.2% (n = 7) were lost to follow-up.

**Conclusion:**

Our findings confirm that nephroblastoma can be effectively treated in resource-constrained settings. Using an approach in which chemotherapy is delivered at a rural-based center by nononcologists and data are used for routine evaluation, care can be delivered in safe, novel ways. Protocol modifications to mitigate chemotherapy toxicities and strong communication between the multidisciplinary team members will likely minimize delays and further improve outcomes in similar settings.

## INTRODUCTION

Nephroblastoma (Wilms tumor [WT]) is the most common pediatric kidney cancer.^1^ With standardized surgical techniques and chemotherapy, survival is greater than 85% in high-income countries.^2^ In contrast, survival rates in African settings are generally less than 45%, although one recent collaborative effort achieved an event-free survival rate of 77%.^3-8^ This outcome disparity reflects lack of access to high-quality, multidisciplinary oncologic care, with many cancer facilities often situated in urban centers and within the private sector.^9-12^

The Butaro Cancer Center of Excellence (BCCOE) in Rwanda is a public-sector facility located in the rural Northern province, providing cancer care in partnership with international nongovernmental organizations and academic cancer institutes.^13^ In this model, clinical care is shifted to general physicians, pediatricians, and internists who receive mentorship from oncology specialists through scheduled conference calls, on-site visits, and e-mail communication. Patients with WT are treated using a nationally approved protocol based on International Society of Pediatric Oncology (SIOP) guidelines and adapted to regionally available resources. Training, supplies (including chemotherapy), social supports for patients, and other resources are supported by the Rwandan Ministry of Health, foundation grants, and individual donations. In this retrospective cohort review, we report outcomes and protocol compliance for WT managed at BCCOE and evaluate delays and deaths at each stage of treatment.

## PATIENTS AND METHODS

### Setting

BCCOE was established in 2012 through a partnership among the Rwandan Ministry of Health, the nonprofit organization Partners in Health/Inshuti Mu Buzima, and the Dana-Farber Brigham and Women’s Cancer Center (DFBWCC). The 160-bed district hospital, a 3-hour drive from the capital Kigali, provides basic imaging (x-ray and ultrasound), laboratory tests, pathology specimen processing, chemotherapy, and social services. Advanced imaging and surgery over the study period were performed at national referral hospitals in Kigali, and pathology reports were provided by pathologists at Brigham and Women’s Hospital, Rwandan referral hospitals, or BCCOE visiting pathologists. Given the lack of radiotherapy available in Rwanda, patients are referred to Mulago Hospital in Uganda on a case-by-case basis and at no cost to the patients. Care is administered regardless of ability to pay.

The care team consisted of Rwandan-trained physicians and nurses without prior specialty training. Additional staff and longitudinal training were provided by Partners in Health/Inshuti Mu Buzima, rotating DFBWCC nurses based at Butaro, and visiting oncologists. This team directed patient care and chemotherapy administration according to the nationally approved protocol (see below). On-site clinicians were supervised by structured weekly phone calls with pediatric oncologists from the DFBWCC with more frequent communication as necessary.

### Patients

This study was approved by ethics review boards in Rwanda (Rwanda National Ethics Committee, Kigali, Rwanda) and the United States (Partners Healthcare, Boston, MA). A retrospective review was performed on 56 consecutive patients with available medical records who were evaluated or treated for nephroblastoma between July 1, 2012, and June 30, 2014, at BCCOE. Data were collected from July 1, 2012, through September 30, 2014. At the time of analysis, three patients were excluded from analysis; two of these patients had more than 50% of their chemotherapy delivered at another facility, and one patient was pathologically diagnosed as having renal clear cell carcinoma. Of the 53 patients remaining, 49 patients initially presented for diagnostic evaluation, and four patients were referred to BCCOE after a nephrectomy. Patients continuing therapy at the end of the study were considered alive and disease free.

### Management and Treatment

Staging, pathology, and treatment were based on the SIOP 2001 guidelines.^14^ Pathology results were classified into low (completely necrotic, cystic partially differentiated), intermediate (regressive, epithelial, stromal, mixed, and focal anaplastic nephroblastoma), and high risk (blastemal, diffuse anaplastic). Treatment was adapted to Rwanda’s health infrastructure and endorsed through expert review at an international conference in Kigali in March 2012 (Data Supplement).

Patients suspected of having nephroblastoma underwent laboratory evaluations (CBCs and renal and liver function tests) and imaging. In compliance with SIOP 2001 recommendations, imaging included abdominal ultrasound, chest x-ray, and computed tomography (CT) scan of the abdomen and pelvis if the ultrasound imaging was suspicious. Pretreatment biopsy was performed only if diagnostic uncertainty existed.

Preoperative chemotherapy depended on tumor stage (a two-cycle regimen of vincristine and dactinomycin for localized stage I to III disease *v* a three-cycle regimen with added doxorubicin for metastatic stage IV and V disease). Preoperative treatment was modified for patients with localized tumors unresponsive to initial therapy; one patient received two additional cycles of chemotherapy, and three patients received an additional three cycles of the metastatic regimen. Surgery was performed at national referral hospitals. Postoperative chemotherapy followed the SIOP higher risk regimen given inconsistent availability of pathology and surgical reports. Radiotherapy is currently not available in Rwanda and thus not part of the national protocol. Basic supportive care including RBC transfusions and intravenous antibiotics was available with intermittent access to platelet transfusions.

### Data Collection and Analysis

Clinical encounters were documented in paper charts and transferred into the OpenMRS (Indianapolis, IN) electronic medical record system. Data were collected by a data collector using a structured chart abstraction form and were analyzed using STATA version 12 (STATA, College Station, TX).

Nonmetastatic, localized disease included stages I to III, and metastatic disease included stages IV and V; this distinction guided the preoperative chemotherapy regimen. Deaths were considered disease related (before treatment began or after relapse) or treatment related (once chemotherapy had been initiated). Loss to follow-up (LTFU) was defined as not returning after surgery or missing the most recent appointment. For assessment of delays, if a patient had a chemotherapy regimen delay, defined as lasting greater than 2 weeks more than the expected duration, all single-event delays were then recorded. Delays during transfer for surgery were defined as greater than 21 days, as recommended by SIOP.^15^

## RESULTS

### Patient Demographics

For the 53 patients included in the study (Table 1), the median age was 3.6 years (interquartile range [IQR], 1.9 to 4.9 years), and 58.5% of patients (n = 31) were girls. The majority of patients came from Rwanda (52 patients, 98.1%) with all five provinces represented, and the remaining patient was from the neighboring country of Burundi (1.9%). Median body mass index at presentation was 15.2 kg/m^2^ (IQR, 14.1 to 16.7 kg/m^2^).

**Table 1:**
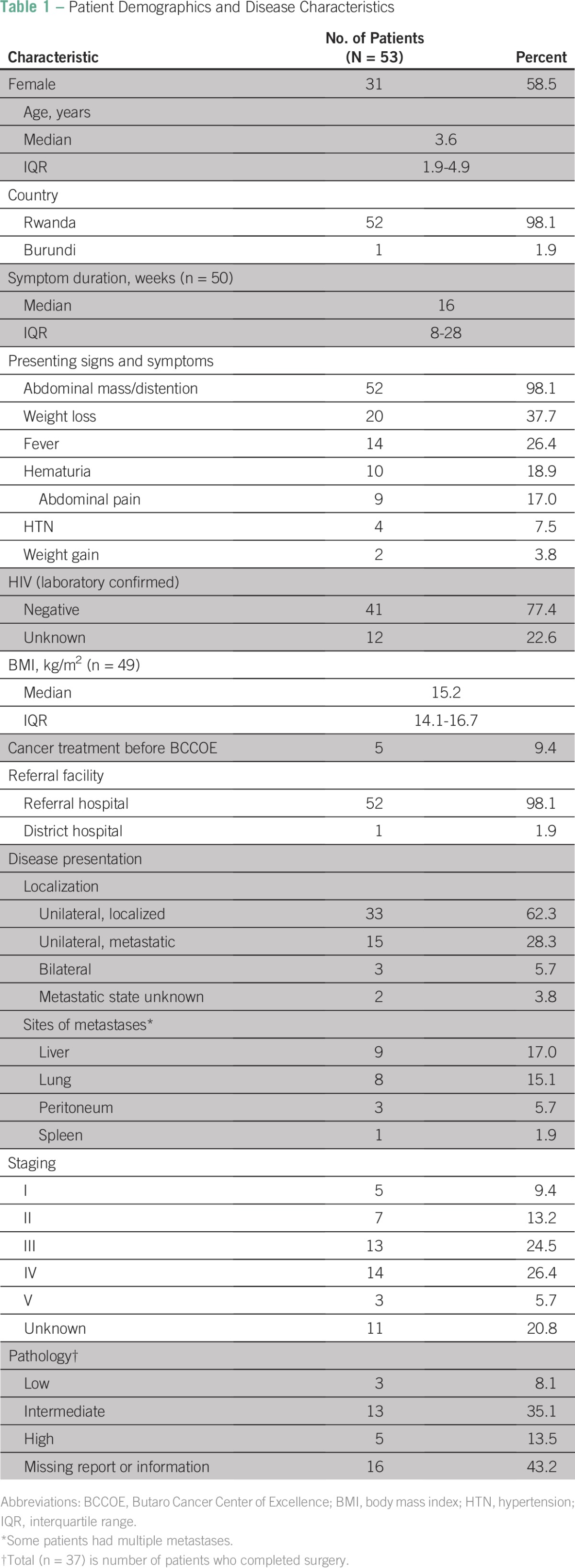
Patient Demographics and Disease Characteristics

### Disease Characteristics

Of the 53 patients, 42 were completely staged (Table 1); five patients (9.4%) had stage I, seven (13.2%) had stage II, 13 (24.5%) had stage III, 14 (26.4%) had stage IV, and three (5.7%) had stage V disease. The remaining patients were not staged as a result of incomplete imaging or missing surgical report (11 patients, 20.8%).Thirty-seven patients completed surgery; three patients (8.1%) had low-risk pathology, 13 (35.1%) had intermediate-risk pathology, and five (13.5%) had high-risk pathology. The remainder had missing or incomplete pathology reports (16 patients, 43.2%).

### Disease Work-Up and Treatment Course

**Fig 1:**
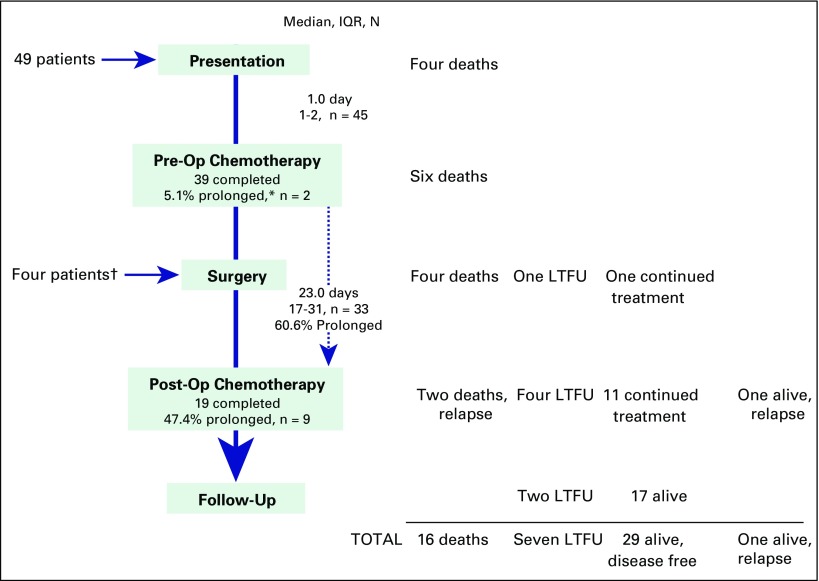
Treatment process and patient events. (*) Prolonged chemotherapy regimen defined as duration at least 2 weeks greater than expected. Prolonged surgical phase was defined as greater than the 21 days, per International Society of Pediatric Oncology recommendations. (†) Did not have preoperative chemotherapy at Butaro Cancer Center of Excellence. Some patients had preoperative chemotherapy but not standardized. IQR, interquartile range; LTFU, loss to follow-up; Post-Op, postoperative; Pre-Op, preoperative.

Forty-nine patients presented for evaluation. Four patients died before beginning treatment; an additional four patients presented after nephrectomy (Fig 1). Median time from patient-reported symptoms to presentation at BCCOE (Table 1) was 16.0 weeks (IQR, 8 to 28 weeks). Patients most often presented with abdominal distention or mass (52 patients, 98.1%), weight loss (20 patients, 37.7%), fever (14 patients, 26.4%), and hematuria (10 patients, 18.9%; Table 1). Forty-seven patients (88.7%) received both chest and abdominal imaging, and six patients (11.3%) had only documented abdominal imaging (ultrasound, CT scan, or magnetic resonance imaging). One patient received a presurgical biopsy. Once evaluated at BCCOE, patients began treatment within a median of 1 day (IQR, 1 to 2 days).

Forty-five patients began preoperative chemotherapy; 39 of these patients completed therapy, and six died during this phase. Patients with nonmetastatic disease completed chemotherapy over a median of 21 days (IQR, 20.5 to 25 days; expected duration of 21 days), and patients with metastatic disease completed treatment over a median of 38 days (IQR, 35 to 48 days; expected duration of 35 days).

After preoperative chemotherapy, the 39 patients were transferred for surgery at national referral centers; 33 of these patients completed surgery. Three patients died during transfer, one died during surgery, one was LTFU, and one was awaiting surgery at the end of the study period. Of 33 patients for whom surgical transfer dates were documented, 60.6% of patients (n = 20) were beyond the 21-day time frame recommended by SIOP.

Including the four patients transferred to BCCOE after surgical resection, 19 of 37 patients completed the 10-cycle high-risk postoperative chemotherapy over a median of 200 days (IQR, 195 to 233 days; expected duration of 182 days); two of these patients were later LTFU. The remaining 18 patients were continuing treatment (n = 11), died after relapse that was clinically noted and verified by imaging (n = 2), were alive with relapse (n = 1), or were LTFU (n = 4). Finally, two patients (one stage III patient and one stage IV patient) received radiotherapy, both of whom remain alive without disease.

### Outcomes

At the end of the study period (Table 2), 30 patients (56.6%) were alive and all but one remained disease free, 16 patients (30.2%) died, and seven patients (13.2%) were LTFU. Of the 17 patients who completed treatment and are in active follow-up, 10 patients were alive at 6 months after completion of their treatment, and five patients were at 1 year. In the case that outcomes are stratified by stage (Data Supplement), it seems that patients with early-stage disease do better, but a fair portion of patients with late-stage WT also do well (Data Supplement); longer term follow-up will be needed to elucidate any relation.

**Table 2:**
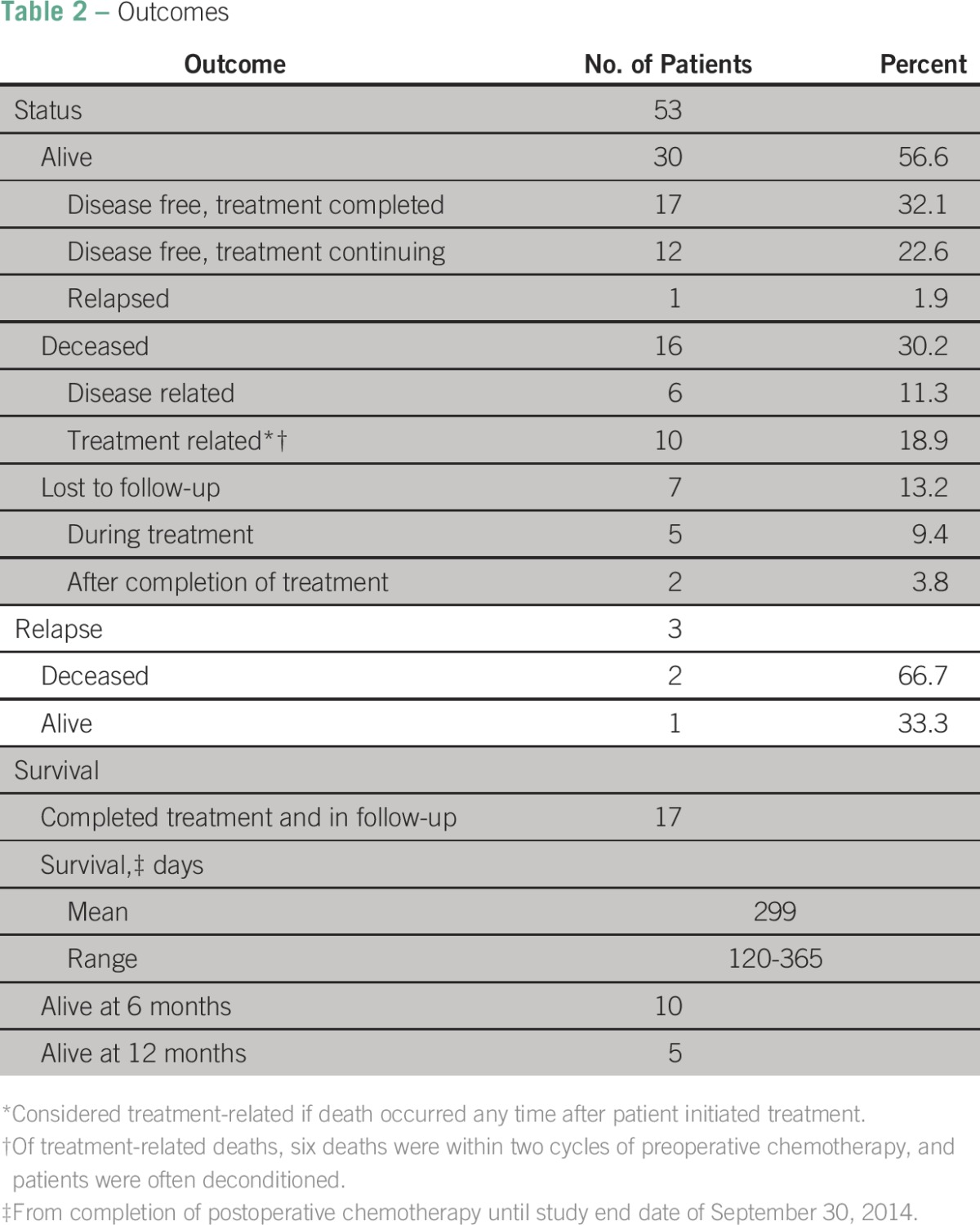
Outcomes

Of the 16 patient deaths, six were classified as disease related (four patients died before initiation of treatment, and two died as a result of relapse on therapy) and 10 were considered treatment related. Of these 10 patients, three had documented evidence of malnutrition, and for the remainder, the cause of death was not clear.

### Delays and Toxicity

Medical delays in therapy (Data Supplement) most commonly were a result of neutropenia, febrile neutropenia, infections, and other laboratory abnormalities (six delays during preoperative chemotherapy and 28 delays during postoperative chemotherapy). Focusing on hematologic toxicity, the incidence of severe grade 4 neutropenia increased with duration of treatment (zero patients at intake to 19 during postoperative chemotherapy).Inadequate patient resources contributed to socioeconomic-driven delays for seven patients who experienced a total of 16 unique delays in care; one delay occurred during preoperative chemotherapy, and 15 occurred during postoperative chemotherapy. Reasons included ill family members and financial or logistical barriers to transportation.

Last, delays arose secondary to limitations of the health care system. Treatment for two patients was delayed after inability to obtain vincristine for preoperative chemotherapy, and delays occurred for three patients as a result of dactinomycin shortages during postoperative chemotherapy. Although radiotherapy is not currently part of the national protocol, 30 patients with confirmed stage III to V disease would have qualified for radiotherapy according to SIOP guidelines, whereas only two patients actually received radiation.

## DISCUSSION

At the BCCOE from July 2012 to June 2014, nephroblastoma represented more than 20% of the pediatric cancers seen and was the most common childhood malignancy.^13^ The incidence is similar to that reported from Zambia and other centers in Africa.^10,16^ This is in contrast to the United States, where WT accounts for only 5% of childhood cancers.^17^ Whether this represents a true difference based on underlying biologic factors or reflects referral and presentation biases is currently unknown. As a result of the slow growth and few symptoms early in the course of WT, these patients may be over-represented because they survive long enough to present for medical evaluation.

More than half of patients presented with advanced disease similar to that seen in other resource-constrained settings.^3,6,18,19^ In contrast, in the United States, only 35% of patients present with stage III or greater disease.^20^ However, staging was imperfect in our study, and the distribution could have been skewed toward either understaging (lack of access to full imaging work-up and incomplete surgical reporting) or overstaging (benign lesions being mistaken for metastases or large tumors mistaken for bilateral tumors on ultrasound). Age and sex distribution were comparable to that seen in similar settings and the United States.^5,6,21^

Our reported outcomes and toxicities for patients with nephroblastoma were encouraging. Although much higher than reported in high-income countries, less than 20% of patients experienced mortality from therapy-related causes, suggesting the current protocol was tolerable for the majority of patients. Treatment-related deaths occurred early in the treatment course during the preoperative chemotherapy and surgery phase. Furthermore, deaths particularly early in treatment were often difficult to distinguish from deaths as a result of disease, given limitations in postmortem documentation. The LTFU rate was 13.2%, which is less than the 20% reported in a Moroccan cohort.^22^ This is perhaps a result of subsidized chemotherapy and social supports at BCCOE^5,6^; however, we currently do not have sufficient data to evaluate contributing factors. Relapse rates may also be probed in the future with additional data.

Although many children successfully completed therapy, there remain challenges in the BCCOE model. The cancer center is situated at a district hospital without an intensive care unit, pediatric surgical services, or ability to conduct advanced radiology (CT and magnetic resonance imaging). As a result, treatment relied on close collaborations and coordination with national referral centers. These changes in care venue contributed to delays seen during surgical transfer. Finally, the lack of oncology specialists based at Butaro Hospital affects care delivery. However, the rates of treatment-related death and LTFU support the use of this model as an interim step toward building national oncology capacity.

Within the BCCOE model, delays arose from medical causes, lack of patient resources, and health system issues; this classification of delays guides subsequent improvements in patient care. Medical delays, including those caused by neutropenia and fever/infection, were the most common and expected in oncologic populations.^18^ More stringent use of dose reductions in underweight and malnourished patients, as recommended by SIOP Pediatric Oncology in Developing Countries Committee,^15,23^ and ongoing education are essential. Medical delays caused by infections could also be addressed through developing capacity to perform blood cultures and broadening antibiotic options.

Malnutrition has been documented among patients with nephroblastoma in similar settings^24^ and likely contributes to medical delays. In our population, mid-upper arm circumference was not standardly recorded,^25^ although body mass index at presentation was similar to a that in a cohort in Malawi.^4^ Increased toxicity during preoperative chemotherapy in malnourished patients with nephroblastoma has been previously addressed with nutritional supplementation.^24^ Protocol modifications, including several rounds of dose-reduced chemotherapy, may also allow patients to medically stabilize before starting full-intensity chemotherapy. At BCCOE, dose reductions for malnutrition were initially not consistently applied. Currently, evaluation by a nutritionist, nutritional support, and dose reductions have begun to be routinely incorporated into care.

In addition to medical causes, lack of patient resources led to care delays in this resource-constrained setting. Location serves as one barrier; some patients travel up to 12 hours for weekly chemotherapy. Social support for transportation will be essential in achieving treatment continuity. In general, emphasis on social services has been cited as critical for treatment of WT in resource-constrained settings.^5,6^ The Rwandan national insurance plan (Muteulle) and BCCOE’s financial support ranging from transport to chemotherapy coverage likely explain the low rate of LTFU at BCCOE compared with similar settings.^4,5,18,22^ Continued emphasis on overcoming barriers to care, such as finances, transportation, and cultural influences not addressed in this work, may further decrease LTFU rates.

The final class of delays stems from the health systems infrastructure. Strengthening communication among health facilities is one improvement initiative. Although existing collaborations among Rwandan referral facilities were central to outcomes, further streamlining care could decrease delays during surgical transfer, which is currently the greatest contributor to delays. Communication again serves a role in timely pathology and surgical reporting. To address missing surgical reports, a standardized intraoperative form was developed in early 2014 to template information required for disease staging and surgical complications. Similar interventions for pathology in addition to cross-institutional tumor boards could further facilitate this communication. Ideally, future increases in multidisciplinary cancer centers in Rwanda will mitigate these delays by centralizing and increasing access to services.

Medication shortages are also a reality that impact patient care in unpredictable ways.^26^ Variations in the demands for chemotherapy, which were subsidized and procured by nongovernmental partners, are challenging to predict, particularly as patient volume increases. Over time, we hope to design a computer-based system to model projected needs. This, in addition to improved communications with the clinical team, will improve the accuracy of drug procurement.

Access to radiotherapy is the final health systems issue discussed and has been a barrier to treatment with curative intent for many cancers seen at BCCOE.^13,27^ On the basis of confirmed clinical staging, an estimated 30 patients in the past 2 years qualified for radiotherapy, which currently costs around US$2,800 per patient. Given its curative and palliative benefits for all cancers, radiotherapy has become a national priority for the Rwandan Ministry of Health.^13^

Beyond achieving clinical outcomes comparable to similar settings, the BCCOE model demonstrates the value of using an implementation science framework. All data collected and analyzed were derived from routine clinical data systems implemented to monitor and improve quality of care. Intentional use of robust data collection tools embedded within delivery systems allows for consistent evaluation without depending on episodic, resource-intensive research efforts. Implementation science is a critical tool in the implementation and improvement of care in settings such as rural Rwanda.

In summary, BCCOE has taken a unique approach to making specialty care accessible in a rural resource-limited setting. Although ultimate outcomes for these patients will require longer follow-up, we show that nephroblastoma can be safely and effectively treated in this setting using this approach. Communication across multidisciplinary teams and multiple facilities was critical, particularly given the rural location of the cancer center. Finally, nephroblastoma protocols may need further adaptations to best serve specific populations. For example, modifications of preoperative chemotherapy in the setting of malnutrition could decrease therapy-related toxicity. Preliminary outcomes suggest nephroblastoma treatment even in resource-constrained settings ought to be a priority given tolerable treatment toxicity and favorable outcomes. Careful documentation of care coupled with a focus on monitoring and evaluation have created the foundation for future improvements. Looking forward, the chemotherapeutic agents used to treat WT have few long-term sequelae, so the majority of children cured of this cancer should be able to resume a normal life and development. Thus, the treatment of WT can serve as a paradigm, demonstrating that a cancer diagnosis is not universally fatal in low-resource settings and children can be successfully treated in the earliest stages of creating a cancer delivery program.
